# Comparison of saliva cotinine and exhaled carbon monoxide concentrations when smoking and after being offered dual nicotine replacement therapy in pregnancy

**DOI:** 10.1111/add.15671

**Published:** 2021-09-20

**Authors:** Bhavandeep Slaich, Ravinder Claire, Joanne Emery, Sarah Lewis, Sue Cooper, Ross Thomson, Lucy Phillips, Darren Kinahan‐Goodwin, Felix Naughton, Lisa McDaid, Miranda Clark, Anne Dickinson, Tim Coleman

**Affiliations:** ^1^ Division of Primary Care University of Nottingham Nottingham UK; ^2^ School of Health Sciences University of East Anglia Norwich UK; ^3^ Division of Epidemiology and Public Health University of Nottingham Nottingham UK

**Keywords:** carbon monoxide, cotinine, dual, nicotine replacement therapy, NRT, pregnancy, smoking, smoking cessation

## Abstract

**Background and Aims:**

Although English Stop Smoking Services routinely offer dual nicotine replacement therapy (NRT) to help pregnant women to quit smoking, little is known about how nicotine and tobacco smoke exposures following this compare with that from smoking. We compared, in pregnant women when smoking and after being offered dual NRT, saliva cotinine and exhaled carbon monoxide (CO) concentrations and numbers of daily cigarettes smoked.

**Design and Setting:**

Secondary analysis of data from three sequential, observational, mixed‐methods cohort studies conducted as part of the Nicotine Replacement Effectiveness and Delivery in Pregnancy programme. Participants were recruited on‐line or in Nottingham University Hospitals (UK) antenatal clinics between June 2019 and September 2020.

**Participants:**

Forty pregnant women, who agreed to try stopping smoking.

**Intervention:**

Participants were offered dual NRT, agreed a smoking quit date and received an intervention to improve adherence to NRT.

**Measurements:**

Saliva cotinine and exhaled CO concentrations and reported number of cigarettes smoked per day.

**Findings:**

There were no differences in saliva cotinine concentrations at baseline and day 7 post quit date [*n* = 20, mean difference = −32.31 ng/ml, 95% confidence interval (CI) = −68.11 to 3.5 ng/ml; *P* = 0.074, Bayes factor = 0.04]. There were reductions in the reported number of cigarettes smoked per day (*n* = 26, mean difference = −7 cigarettes, 95% CI = −8.35 to −5.42 cigarettes, *P* < 0.001) and concurrently in exhaled CO concentrations (*n* = 17, ratio of geometric means = 0.30 p.p.m., 95% CI = 0.17–0.52 p.p.m.; *P* < 0.001).

**Conclusion:**

Pregnant women who smoke and are offered dual nicotine replacement therapy (NRT) appear to show no change in their exposure to cotinine compared with their pre‐NRT exposure levels but they report smoking fewer cigarettes, as validated by reductions in exhaled carbon monoxide concentrations.

## INTRODUCTION

Smoking during pregnancy is the largest modifiable risk factor for morbidity and mortality for both the mother and developing fetus [1, 2]. It is causally associated with fetal growth restriction and is also associated with other pregnancy complications, including stillbirth, prematurity and miscarriage [3, 4]. Parental smoking is a significant risk factor for smoking uptake in young people and smoking is a major risk factor for six of the eight leading causes of global mortality [5, 6]. Despite this, pregnant women continue to smoke, with the proportion being 12 and 13% in the United Kingdom and United States, respectively [7, 8]. Actual smoking rates are probably higher due to stigma causing an under‐reporting of smoking [9].

UK smoking cessation guidelines recommend offering nicotine replacement therapy (NRT) to pregnant women who smoke if behavioural support has been ineffective [10], with guidance globally recommending similar approaches [11, 12, 13]. NRT is considered safer than smoking because nicotine is provided without the toxins found in cigarettes, such as tar and carbon monoxide (CO) [14]. Routine UK practice involves offering dual NRT as a first‐line smoking cessation treatment [15].

NRT is an effective smoking cessation tool outside pregnancy, with dual NRT more effective than mono NRT [16]. NRT may be less effective in pregnancy, but there is some uncertainty surrounding this finding [17]. One hypothesis which may partially explain the apparent reduced efficacy of NRT in pregnancy is that in pregnancy there is acceleration of nicotine metabolism caused by an induction of nicotine metabolizing enzymes [18, 19, 20]. Consequently, for any given dose, NRT will generate a lower nicotine blood concentration in pregnant women. Such concentrations may be insufficient to alleviate smoking withdrawal symptoms and provide therapeutic benefits. To our knowledge, randomized controlled trials (RCT) investigating the use of NRT as a smoking cessation pharmacotherapy during pregnancy have almost all used a mono NRT regime [17, 21]. It may be that the dose of NRT offered in mono NRT treatment regimes is insufficient.

Using dual rather than mono NRT in routine care could help more pregnant women to quit smoking; observational analyses of data from English Stop Smoking Services found being prescribed dual NRT during pregnancy was strongly associated with smoking cessation, whereas there was no association with mono NRT [22]. Women's acceptance and use of NRT can be limited by concerns about fetal harms caused by nicotine [23]. Although such concerns are valid and real to those that hold them, they are very probably unfounded; there is no evidence that nicotine used as NRT instead of smoking causes fetal harm [24]. More information regarding nicotine exposure following dual NRT and after smoking could help to reassure pregnant women about the extent of nicotine exposure when offered dual NRT, but this has so far only been investigated in a cohort of five women [25]. This study therefore aims to compare changes in the indicators of smoking intensity and nicotine exposure in pregnant women when they smoke with those after being offered dual NRT. To achieve this aim, we will compare: (1) cotinine concentrations when smoking with those measured after being offered dual NRT; (2) exhaled CO concentrations when smoking with those measured after being offered dual NRT; and (3) the number of daily cigarettes reported as being smoked, when smoking only and after being offered dual NRT.

## METHODS

### Design

This is a secondary analysis of data from three sequential cohort studies in which a prototype intervention intended to improve pregnant women's NRT adherence was piloted on pregnant smokers. Cohorts were part of a larger programme, Nicotine Replacement Effectiveness and Delivery in Pregnancy (N‐READY), which aims to test whether the intervention affected NRT adherence and smoking cessation. Ethical approval was given by the Nottingham 1 Research Ethics Committee.

**FIGURE 1:**
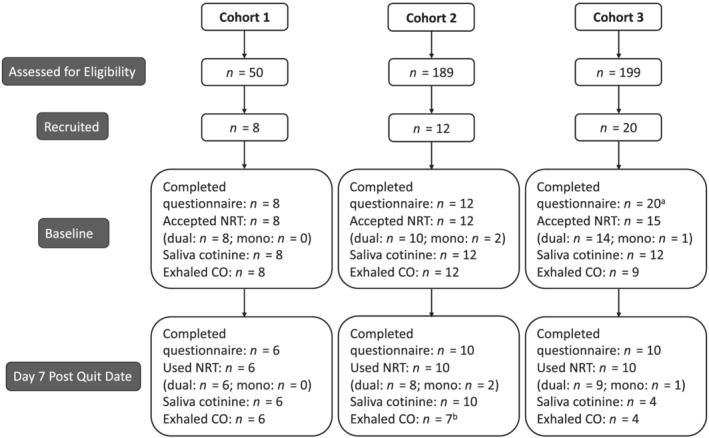
Consolidated Standards of Reporting Trials (CONSORT) diagram illustrating the number of participants and data collection at each stage of the study. ^a^Five of 20 women in cohort 3 dropped out after completing the baseline questionnaire and did not receive a stop smoking consultation/offer of NRT; ^b^three of 10 women in cohort 2 could not give a carbon monoxide (CO) sample due to COVID‐19 restrictions on face‐to‐face contact—CO samples were carried out remotely in cohort 3

Data were collected at baseline, when participants were smoking and then women were offered dual NRT to start using on an agreed quit date. Data were again collected 7 days post quit date. Figure 1 illustrates the study design and n‐numbers at each stage. The pre‐registered research aims and analysis plan can be found at https://osf.io/37t4s/

### Participants

Women were eligible if pregnant and with less than 25 weeks’ gestation, aged over 16 years, smoked at least 10 cigarettes per day pre‐pregnancy and currently smoking, interested in receiving smoking cessation support, willing to try NRT and to set a quit date within 14 days. Women with less than 25 weeks’ gestation were enrolled because the original cohort studies piloted study methods, and hence inclusion criteria, to be used in a future RCT to test the efficacy of the intervention being optimized in the cohorts. A gestation limit was specified because an outcome of the RCT will be a measure of prologued smoking cessation between a point earlier in pregnancy and childbirth, and so recruiting women before 25 weeks ensures that the period of smoking cessation monitored would be sufficient to be clinically meaningful. Participants were required to be able to give consent, understand English and own a smartphone, because the study intervention and data collection required participants to open links to websites sent via text. Women already enrolled into a cessation study or using NHS stop smoking support were excluded.

Eligible women were identified at Nottingham University Hospitals Trust antenatal ultrasound and outpatient clinics. In the third cohort women were also identified through Facebook advertisements, a change necessitated by COVID‐19 restrictions on face‐to‐face recruitment. Recruitment was initially planned to run from 1 June 2019 to 31 March 2020. Due to the first UK lockdown of 2020, collection of exhaled CO samples initially ceased. In the third cohort only, following lockdown, all data and samples were collected remotely, and data collection was completed by September 2020.

### Intervention

Participants were offered dual NRT and behavioural support from trained practitioners. The dual NRT comprised a choice of a daily nicotine patch (e.g. Nicorette 16‐hour 15 or 25 mg; NiQuitin 24‐hour 14 or 21 mg) combined with a choice of short‐acting NRT (e.g. Nicorette Cools Lozenges (2 or 4 mg), Nicorette inhalator (15 mg) or Nicorette QuickMist mouth spray). Participants were encouraged to use as much NRT as required to ameliorate symptoms of withdrawal and were advised to continue using NRT during smoking lapses of fewer than 14 days, provided they still aimed to quit smoking. To increase NRT adherence, participants received a prototype intervention integrated into routine NHS support comprising telephone consultations, leaflets, websites and text messages designed to reinforce NRT adherence.

### Measures

#### Baseline

Researchers collected data including date of birth, ethnicity, qualifications, gestation and whether women had a partner who smoked. Researchers asked the number of cigarettes smoked daily, use of e‐cigarettes, smoking in previous pregnancies and how soon after waking women smoked their first cigarette. In cohort 3, some participants declined to give an exact number of cigarettes smoked daily and instead reported a range (e.g. 8–10 cigarettes).

Researchers obtained saliva samples to measure cotinine and anabasine concentrations and an exhaled CO reading. Cotinine is a proxy for nicotine exposure from any source, anabasine is a proxy for nicotine exposure from tobacco smoke and exhaled CO is a proxy for tobacco smoke exposure. In cohorts 1 and 2, after seeking baseline information and samples at a face‐to‐face consultation, the same researchers immediately delivered study smoking cessation interventions. In cohort 3, questions were asked via telephone and researchers sent out equipment for women to provide saliva and CO samples remotely and arranged appointments with different research team members for remote intervention delivery.

#### Day 7 post quit date

Using methods particular to each cohort described above we sought saliva and exhaled CO samples. We asked whether participants had smoked in the past week and, if currently smoking, the number of cigarettes per day. We also asked about use of NRT in the intervening 7 days.

Saliva samples were obtained from each participant using clean salivettes. This involved the placement of a sterile swab under the participant's tongue for 5 minutes, and then placing the swab into a sterile, labelled vessel. In cohorts 1 and 2, saliva samples were obtained by researchers and in cohort 3 samples were obtained by participants themselves. Samples were posted to ABS Laboratories (York, UK) and were analysed in a single batch; cotinine and anabasine levels were quantified using a standard immunoassay technique. In cohorts 1 and 2, CO concentration measurements were obtained by researchers using a hand‐held CO monitor and in cohort 3 we sent a CO monitor to each participant and advised them remotely on how to use the monitors (iCO Smokerlyzer®; Bedfont Scientific, Kent, UK).

### Analyses

For baseline data, continuous measures are presented as means with standard deviations (SD). Categorical measures are presented using frequencies and percentages. χ^2^ tests and *t*‐tests were used for discrete and continuous variables, respectively, to assess differences between those who provided both baseline and follow‐up cotinine samples and those who did not. Baseline and follow‐up data of salivary cotinine, exhaled CO and number of cigarettes smoked per day is presented as means with SDs. Individual saliva cotinine and exhaled CO concentrations and number of cigarettes smoked per day at baseline and at day 7 post quit date are presented as line graphs. Histograms are used to summarize the magnitude of change in these three variables from baseline to day 7 post quit date. Analyses were conducted on the whole cohort, within those reporting smoking abstinence and who had no detectable anabasine at follow‐up and within those who reported continued smoking and/or had detectable anabasine in their follow‐up saliva sample. Hence, anabasine was used to confirm smoking abstinence. A natural log‐transformation of exhaled CO concentrations was used to achieve a normal distribution. Paired *t*‐tests were used to assess ‘within‐participant’ differences in saliva cotinine, exhaled CO and number of cigarettes smoked per day at baseline when smoking and day 7 post quit date after being offered dual NRT (alpha 0.05). For exhaled CO, we present the back‐transformed estimates of the baseline and follow‐up differences, which is the ratio of the geometric means. Analyses were conducted using Stata version 15.

For the six cohort 3 participants who reported a range instead of an integer for the ‘number of cigarettes smoked per day (CPD)’, in the main analysis the lower bound of this range was imputed at baseline and the higher bound at follow‐up. For example, a participant who reported smoking 8–10 CPD at baseline had this imputed as eight daily cigarettes at baseline, and if the same range was reported at follow‐up, 10 was imputed. This was a conservative assumption, but we also report a sensitivity analysis using the upper bound at baseline and the lower bound at follow‐up.

Upon analysis completion, we calculated Bayes factors from the differences in saliva cotinine in the whole cohort, saliva cotinine in abstinent women and exhaled CO in non‐abstinent women [26]. An expected difference of 141 ng/ml in saliva cotinine concentration for the whole cohort was taken from a study of pregnant women using dual NRT [25]. A meta‐analysis comparing nicotine exposure during pregnancy when smoking and when abstinent with NRT provided an expected difference of 75.3 ng/ml in saliva cotinine concentration in abstinent women [27]. An expected difference of 3 parts per million (p.p.m.) in exhaled CO concentration was taken from a study of pregnant women using NRT while currently smoking [28]. A conservative approach for estimation using a half‐normal distribution was used, in which the SD is equal to the expected effect size.

## RESULTS

Figure 1 shows that 50, 189 and 199 (438 total) women were assessed for eligibility to join cohorts 1, 2 and 3, respectively. Forty participants joined the study and provided baseline demographic data, with eight, 12 and 20 women recruited into cohorts 1, 2 and 3, respectively. Thirty‐five (87.5%) participants were issued with NRT, of whom 32 accepted dual NRT. Valid baseline saliva samples were provided by 32 (80%) participants and valid baseline expired CO samples by 29 (72.5%). At day 7 post quit date, 20 (62.5%), 17 (58.62%) and 26 (65%) women provided a follow‐up saliva sample, an exhaled CO reading and reported a number of cigarettes smoked per day, respectively. Twenty (50%) participants provided paired saliva samples and 26 (65%) participants reported using NRT in the first 7 days after the quite date. At day 7 post quit date, 15 (58%) participants reported smoking abstinence, with eight validated by the absence of anabasine in saliva.

Table 1 shows the characteristics of women who provided both baseline and follow‐up saliva samples (*n* = 20) and those who did not (*n* = 20). Those who provided both samples had significantly lower baseline exhaled CO and saliva cotinine concentrations and were less likely to have smoked in previous pregnancies, suggesting that women with higher nicotine dependence dropped out of treatment and therefore out of the study. Of the 20 women with paired samples, 20 (100%) were issued dual NRT and 20 (100%) reported using NRT in the 7 days following the quit date.

**TABLE 1:** Participant baseline characteristics; *n* (%) or mean (SD).

Characteristic	Provided saliva samples at baseline and at follow‐up (*n* = 20)	Failed to provide saliva samples at baseline and at follow‐up (*n* = 20)
Age (years)	29.05 (4.89)	29.25 (5.53)
Ethnicity		
White	20 (100%)	18 (90%)
Qualifications^d^		
None	1 (5%)	4 (20%)
GCSEs	13 (65%)	6 (30%)
A‐levels	6 (30%)	6 (30%)
Degree	0 (0%)	4 (20%)
Gestation (days)	110.5 (38.28)	99.3 (28.28)
Partner who smokes		
No partner	2 (10%)	4 (20%)
Partner who smokes	14 (70%)	11 (55%)
Partner is a non/ex‐smoker	4 (20%)	5 (25%)
Smoking status in previous pregnancies		
No previous pregnancies	1 (5%)	2 (10%)
Yes	16 (80%)	17 (85%)
No	3 (15%)	1 (5%)
Number of hours after waking before first cigarette		
Within 5 minutes	5 (25%)	6 (30%)
6–30 minutes	10 (50%)	10 (50%)
31–59 minutes	2 (10%)	2 (10%)
1–2 hours	2 (10%)	2 (10%)
More than 2 hours	1 (5%)	0 (0%)
E‐cigarette use	1 (5%)	1 (5%)
Number of cigarettes smoked per day^c^	8 (3.63)	11 (6.58)
Saliva cotinine concentration (ng/ml)	156.54 (69.13)	164 (103.87)
Exhaled carbon monoxide concentration (p.p.m.)	14 (7.24)^a^	23.67 (11.14)^b^

^a^ *n* = 17;

^b^ *n* = 12.

^c^ Data have been rounded to the nearest cigarette;

^d^ GCSEs (General Certificate of Secondary Education) are sat at age 16; A (Advanced) levels are sat at age 18 and degree refers to any undergraduate degree certificate.

**FIGURE 2:**
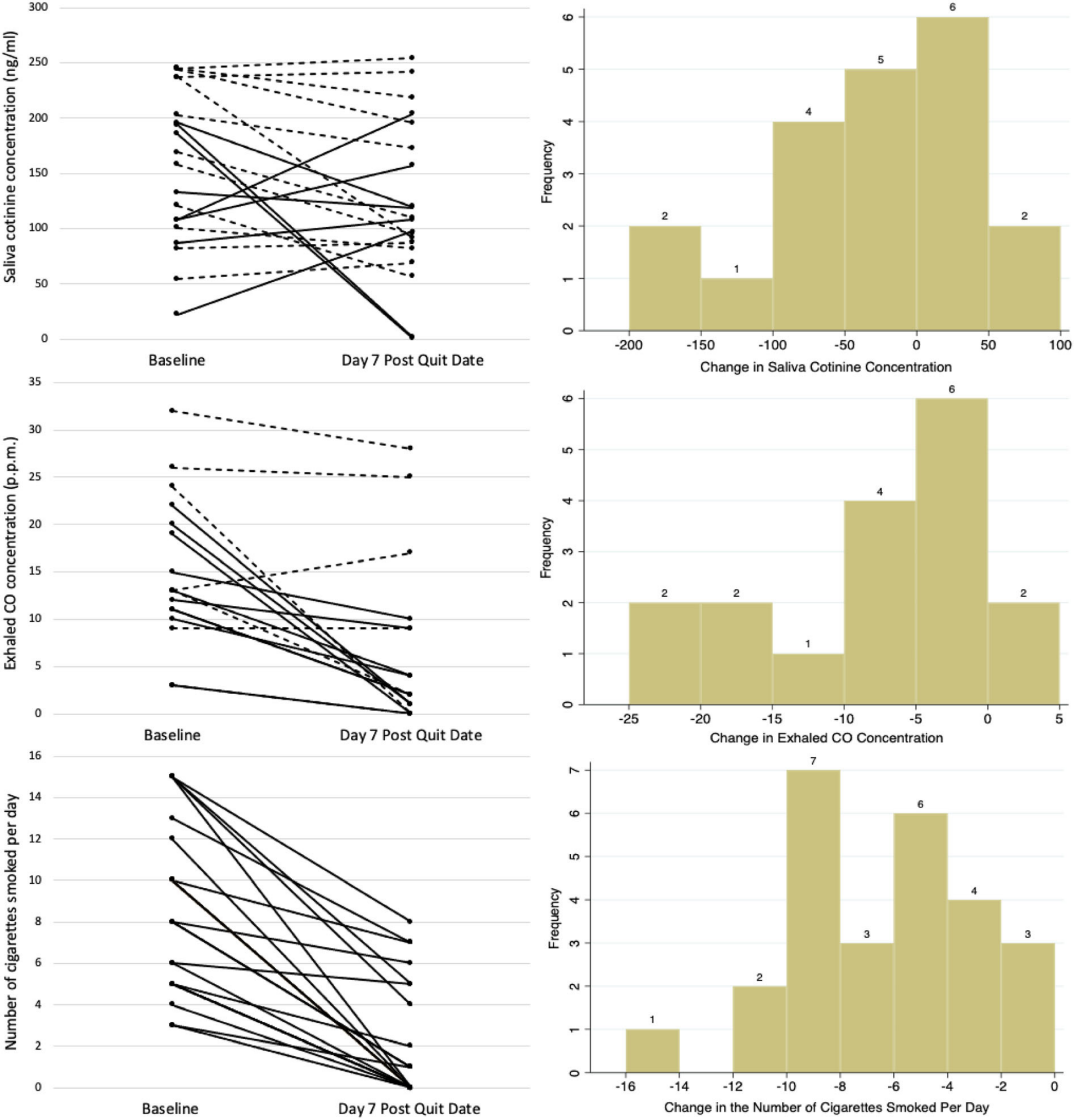
Left: a visual representation of individual saliva cotinine concentration, exhaled carbon monoxide (CO) concentration (hatched lines represent participants who reported smoking at day 7 post quit date and continuous lines represent abstinent participants) and number of cigarettes smoked per day (no hatching of lines) at baseline and at day 7 post quit date. Right: histograms summarizing the magnitude in changes of saliva cotinine concentration, exhaled CO concentration and number of cigarettes smoked per day from baseline and day 7 post quit date

Figure 2 visually represents participants’ changes in saliva and CO concentrations and in the reported number of cigarettes smoked per day between baseline and day 7 post quit date. Hatched lines represent participants who reported smoking at follow‐up and continuous lines represent abstinent participants. Twelve (60%) participants showed reductions in saliva cotinine concentrations; 15 (88%) participants demonstrated a reduction in exhaled CO concentration and all reported smoking fewer cigarettes; histograms summarize the magnitude of changes.

Table 2 compares the indicators of smoking intensity at baseline and day 7 post quit date for all participants. There was no significant difference between the mean saliva cotinine concentration at baseline and at day 7 post quit date [*n* = 20, mean difference = −32.31 ng/ml, 95% confidence interval (CI) = −68.11 to 3.50 ng/ml; *P* = 0.074, Bayes factor = 0.04]. There was a significant reduction in the mean exhaled CO concentration (*n* = 17, ratio of geometric means = 0.30 p.p.m., 95% CI = 0.17 to 0.52 p.p.m.; *P* < 0.001) and mean number of cigarettes smoked per day (*n* = 26, mean difference = −7 cigarettes, 95% CI = −8.35 to −5.42 cigarettes, *P* < 0.001) between baseline and at day 7 post quit date. The sensitivity analysis which used alternative range bounds for the number of cigarettes smoked per day variable did not produce materially different findings.

**TABLE 2:** Baseline to day 7 post quit date ‘within participant’ differences in indicators of smoking intensity.

	Baseline mean (SD)	Day 7 post quit date mean (SD)	Mean difference (95% CI)	*P*‐value
Saliva cotinine (ng/ml) (*n* = 20)	156.54 (69.13)	124.23 (72.06)	−32.31 (−68.11, 3.50)	0.074
Exhaled carbon monoxide^a^ (p.p.m.) (*n* = 17)	13.02	3.21	0.30 (0.17, 0.52)	< 0.001
Number of cigarettes smoked per day^b^ (*n* = 26)	9 (3.86)	2 (2.73)	−7 (−8.35, −5.42)	< 0.001^c^

Paired *t*‐tests were used to compare the differences between baseline and day 7 post quit date means. SD = standard deviation; CI = confidence interval; p.p.m. = parts per million.

^a^ Back‐transformed exhaled carbon monoxide data. Means represent geometric means. Mean differences presented as ratio of geometric means.

^b^ Data have been rounded to the nearest cigarette;

^c^ sensitivity analysis confirmed this finding.

Table 3 shows the same comparisons as Table 2 by validated smoking status at day 7 post quit date. In the abstinent women, the mean reduction in saliva cotinine was not statistically significant (*n* = 8, mean difference = −28.30 ng/ml, 95% CI = −122.27 to 65.68 ng/ml; *P* = 0.500, Bayes factor = 0.30); however, the mean reduction in exhaled CO concentration (*n* = 5, ratio of geometric means = 0.20 p.p.m., 95% CI = 0.07–0.57 p.p.m.; *P* = 0.013) was statistically significant. In continuing smokers the mean reductions in saliva cotinine (*n* = 12, mean difference = −34.98 ng/ml, 95% CI = −63.75 to −6.22 ng/ml; *P* = 0.022) and number of cigarettes smoked per day (*n* = 12, mean difference = −5 cigarettes, 95% CI = −7.57 to −3.26 cigarettes; *P* = 0.002) were statistically significant, although the mean reduction in exhaled CO concentration (*n* = 8, ratio of geometric means = 0.65 p.p.m., 95% CI = 0.37–1.13; *P* = 0.106, Bayes factor = 0.18) was not statistically significant.

**TABLE 3:** Subgroup analysis: baseline to day 7 post quit date ‘within‐participant’ differences in indicators of smoking intensity in pregnant smokers by smoking status at day 7 post quit date.

	Reported smoking abstinence and no detectable saliva anabasine at follow‐up			
	Baseline mean (SD)	Day 7 post quit date mean (SD)	Mean difference (95% CI)	*P*‐value
Saliva cotinine (ng/ml) (*n* = 8)	129.29 (60.91)	100.99 (70.07)	−28.30 (−122.27, 65.68)	0.500
Exhaled air carbon monoxide^a^ (p.p.m.) (*n* = 5)	9.49	1.14	0.20 (0.07, 0.57)	0.013

	Reported continued smoking and/or detectable saliva anabasine at follow‐up			
	Baseline mean (SD)	Day 7 post quit date mean (SD)	Mean difference (95% CI)	*P*‐value
Saliva cotinine (ng/ml) (*n* = 12)	174.71 (70.66)	139.73 (72.05)	−34.98 (−63.75, −6.22)	0.022
Exhaled air carbon monoxide^a^ (p.p.m.) (*n* = 8)	12.97	8.04	0.65 (0.37, 1.13)	0.102
Number of cigarettes smoked per day^b^ (*n* = 12)	9 (4.02)	4 (3.03)	−5 (−7.57, −3.26)	0.002^c^

Paired *t*‐tests were used to compare differences between baseline and day 7 post quit date means. SD = standard deviation; CI = confidence interval; p.p.m. = parts per million.

^a^ Back‐transformed exhaled carbon monoxide data. Means represent geometric means. Mean differences presented as ratio of geometric means;

^b^ data have been rounded to the nearest cigarette;

^c^ sensitivity analysis confirmed this finding.

## DISCUSSION

Our findings demonstrate that pregnant women who reported using dual NRT had similar saliva cotinine concentrations and therefore nicotine exposures as when smoking, but reported smoking substantially fewer daily cigarettes and concurrent reductions in exhaled CO concentrations validated the reports. Additionally, pregnant women who continued to smoke while concurrently using dual NRT showed a reduction in both saliva cotinine concentrations and the number of cigarettes smoked per day, but no change in exhaled CO concentrations.

A study limitation is the small sample size and consequent low statistical power limiting the number of explanatory analyses which could be performed. Another limitation concerns the validity of ‘cigarettes per day’ data. Some participants reported a range rather than a number; however, we believe that imputed values used resulted in a conservative analysis, findings of which are probably valid. Findings from a sensitivity analysis which used alternative imputation assumptions were similar, and concurrent reductions in exhaled CO concentrations, support the notion that reported differences in smoking intensity before and after being offered dual NRT are probably valid. A further limitation is the unclear timing of samples with respect to exposures, and cotinine and CO concentrations would fall as time elapsed after smoking or using short‐acting NRT. However, despite the half‐lives of saliva cotinine and exhaled CO being 17 and 5–6 hours, respectively, ‘spot’ saliva cotinine and exhaled CO measures can detect exposures occurring up to 1 week (saliva cotinine) and 48 hours (CO) previously. Hence, with the relatively short follow‐up used, we believe findings are probably valid. Although sample collection was more difficult than anticipated and the study smaller than planned, behavioural changes were marked, so there was still sufficient power to detect these. Additionally, although more heavily nicotine‐addicted women failed to return for study treatments, dropout rates were similar to those in routine care to which findings generalize most readily. Finally, this study could be criticized for having researchers collect saliva and exhaled CO samples and then, for cohort 3, using remote sample collection procedures due to restrictions imposed by the COVID‐19 pandemic. We do not feel this is significant, as remote collection of saliva samples is a widely accepted practice and one which our research group has previously conducted. Unfortunately, only four paired exhaled CO samples were received when CO monitors were sent to participants for remote sample collection; however, it is probable these measurements were collected reliably, as participants were taught the correct processes [29, 30].

To our knowledge, this is the largest study to investigate nicotine exposure and smoking behaviour in pregnant women who have been offered dual NRT and the only attempt to validate reported changes in smoking behaviour and tobacco smoke exposure with exhaled CO concentration measurement. We also validated participants’ claims of smoking cessation with measures of saliva anabasine concentration. Comparison of within‐participant measurements is a study strength. However, with this design, participant characteristics which change between samples being taken could influence findings. One such factor is nicotine metabolism, which accelerates in early pregnancy, although it is unlikely that this would change enough in 7 days to have marked effects. Additionally, increased nicotine metabolism would not explain the reductions in the number of cigarettes smoked and CO concentrations. Another study strength lies in the availability of some data on NRT adherence, permitting analyses within those reporting NRT use.

Only one small study has investigated similar questions [25]. This compared saliva cotinine and exhaled CO concentrations in five of 327 (1.53%) pregnant trial participants when smoking and after being offered dual NRT as 15‐mg/16‐hour nicotine patches and 2‐mg gum, and demonstrated reduction in saliva cotinine concentrations (mean difference = −141, 95% CI = −47 to −236) [25]. Exhaled CO concentrations were reported as also lower, but no formal statistical comparisons were made and adherence to NRT was not monitored [25]. Our study builds upon this work by demonstrating that women who have reported using dual NRT were exposed to less tobacco smoke. Although, in our study, saliva cotinine concentrations were lower following the use of dual NRT, these differences were neither as marked nor were they statistically significant. This could be because, in the Hegaard study, women were instructed to stop NRT if they smoked at all, whereas we encouraged NRT use during smoking lapses. Our findings are consistent with a secondary analysis of RCT data, which found that in pregnant smokers, compared to when smoking, pregnant women using patch NRT had no greater nicotine exposure but smoked less [28]. Similarly, a systematic review found that, compared to when smoking, pregnant women using mono NRT and abstinent from smoking had reduced saliva cotinine concentrations [27].

Importantly, this study sought women motivated to stop smoking, principally from usual care settings, and study interventions were combined with usual cessation care, such that participants probably found these indistinguishable from routine NHS smoking cessation care. Additionally, after offering NRT, we used unobtrusive study measures which are unlikely to have unduly affected participants’ behaviours and we offered study NRT in a manner consistent with NHS cessation care. Hence, study findings are generalizable to pregnant women who smoke and accept smoking cessation support.

## CONCLUSION

This study was conducted to address concerns of pregnant women regarding nicotine exposure while using NRT in pregnancy. Compared to when smoking, pregnant women who are offered dual NRT show no increase in their exposure to nicotine, smoke less and exhale less CO, meaning their exposure to cigarette smoke toxins is reduced. Even within pregnant women who admitted concurrent smoking we found, compared to smoking, an offer of dual NRT resulted in reduced nicotine exposure.

## DECLARATION OF INTERESTS

None.

## AUTHOR CONTRIBUTIONS

**Bhavandeep Slaich:** Conceptualization; formal analysis; methodology; project administration; software; visualization. **Ravinder Claire:** Conceptualization; formal analysis; methodology; software; supervision. **Joanne Emery:** Conceptualization; formal analysis; methodology; software; supervision. **Sarah Lewis:** Data curation; formal analysis; methodology. **Sue Cooper:** Writing‐review & editing‐Supporting. **Ross Thomson:** Writing‐review & editing‐Supporting. **Lucy Phillips:** Writing‐review & editing‐Supporting. **Darren Kinahan‐Goodwin:** Writing‐review & editing‐Supporting. **Felix Naughton:** Writing‐review & editing‐Supporting. **Lisa McDaid:** Writing‐review & editing‐Supporting. **Miranda Clark:** Writing‐review & editing‐Supporting. **Anne Dickinson:** Writing‐review & editing‐Supporting. **Tim Coleman:** Conceptualization; data curation; funding acquisition; investigation; methodology; resources; supervision.
